# Investigation of mosquito larval habitats and insecticide resistance in an area with a high incidence of mosquito-borne diseases in Jining, Shandong Province

**DOI:** 10.1371/journal.pone.0229764

**Published:** 2020-03-04

**Authors:** Yang Wang, Peng Cheng, Boyan Jiao, Xiao Song, Haiyang Wang, Haifang Wang, Huaiwei Wang, Xiaodan Huang, Hongmei Liu, Maoqing Gong

**Affiliations:** 1 Shandong Institute of Parasitic Diseases, Shandong First Medical University & Shandong Academy of Medical Sciences, Jining, Shandong Province, China; 2 School of Medicine and Life Sciences, University of Jinan-Shandong Academy of Medical Sciences, Jinan, Shandong Province, China; 3 Jining Center for Disease Control and Prevention, Jining, Shandong Province, China; Montana State University, UNITED STATES

## Abstract

**Background:**

To investigate mosquito larval habitats and resistance to common insecticides in areas with high incidence rates of mosquito-borne diseases in Jining, Shandong Province, and to provide a scientific basis for the future prevention and control of mosquito-borne diseases and the rational use of insecticides.

**Methods and results:**

From June to September 2018, mosquito habitat characteristics and species compositions in Jintun town were studied through a cross-sectional survey. Larvae and pupae were collected in different habitats using the standard dipping technique. A total of 7,815 mosquitoes, comprising 7 species from 4 genera, were collected. Among them, *Culex pipiens pallens* (n = 5,336, 68.28%) was the local dominant species and found in all four habitats (rice paddies, irrigation channels, water containers, drainage ditches). There were 1,708 *Cx*. *tritaeniorhynchus* (21.85%), 399 *Anopheles sinensis* (5.11%), 213 *Armigeres subalbatus* (2.72%), 124 *Aedes albopictus* (1.59%), and 35 other (*Cx*. *bitaeniorhynchus* and *Cx*. *halifaxii*) (0.45%) mosquito samples collected. Spearman correlation analysis was employed to evaluate the relationship between larval density and the physicochemical characteristics of the breeding habitat. It was found that the larval density of *Cx*. *tritaeniorhynchus* correlated positively with water depth (*r* = 0.927 *p* = 0.003), the larval density of *An*. *sinensis* correlated positively with dissolved oxygen (DO) (*r* = 0.775 *p* = 0.041) and the larval density of *Cx*. *p*. *pallens* correlated positively with ammonia nitrogen (*r* = 0.527 *p* = 0.002). Resistance bioassays were carried out on the dominant populations of *Cx*. *p*. *pallens*: mosquitoes presented very high resistance to cypermethrin and deltamethrin, moderate resistance to dichlorvos (DDVP), and low resistance to *Bacillus thuringiensis israelensis* (*Bti*), with decreased susceptibility to propoxur.

**Conclusion:**

We showed that mosquito species vary across habitat type and that the mosquito larval density correlated positively with certain physicochemical characteristics in different habitats. In addition, *Cx*. *p*. *pallens* developed different levels of resistance to five insecticides. Vector monitoring should be strengthened after an epidemic, and further research should be conducted to scientifically prevent and kill mosquitoes.

## Introduction

Mosquitoes not only bite and harass humans but also transmit a variety of diseases, such as filariasis, dengue fever, malaria and epidemic encephalitis [1–3]. According to the national legal infectious disease epidemic situation report of the Bureau of Disease Control and Prevention, there were no cases of filariasis infection in China in 2017; however, the numbers of cases of dengue fever, malaria and epidemic encephalitis were 5,893, 2,697 and 1,147, with 2, 6 and 79 deaths, respectively. *Culex pipiens pallens* is the most abundant mosquito species in the Jining area of Shandong Province of northern China [4,5]; it can transmit Bancroftian filariasis and is also a potential vector of West Nile virus (WNV), both of which seriously endanger human health [6,7]. Although the Jining area was once highly endemic for filariasis, malaria and epidemic encephalitis [8–10], these diseases have been controlled or eliminated by comprehensive prevention and control practices implemented over decades. However, in recent years, the rapid development of the social economy and the increase in travel to heavily endemic areas has resulted in imported infections; thus, the prevalence of mosquito-borne diseases is increasing due to population movements [2,3,11]. In August of 2017, the first outbreak of dengue fever occurred in Jining city, Jiaxiang County; a total of 79 cases were reported, and the local epidemic was caused by imported cases [3]. Chemical insecticides, which produce a rapid effect and have a long duration of action, are convenient for use. Unfortunately, the long-term use of chemical insecticides has resulted in high resistance, especially in *Cx*. *p*. *pallens* [5,12]. Therefore, it is necessary to develop more effective strategies to control mosquitoes, consequently reducing the risk of transmission of mosquito-borne diseases.

To control the number of mosquitoes and prevent the spread of mosquito-borne diseases, it is necessary to understand the life history and living habits of mosquitoes and to inhibit mosquito breeding by eliminates the environments in which they develop [13–15]. Mosquitoes develop in four stages, namely, the egg, larval, pupae and adult stages, and the first three stages are completed in water [16,17]. Therefore, aquatic habitats are important for mosquito breeding. Larval source management (LSM) involves the management of water bodies that are potential larval habitats to prevent the development of immature mosquitoes into adults [18,19], and studies have shown that mosquito LSM has an important potential impact on mosquito-borne disease transmission both in urban and rural areas [20–22]. For example, in urban areas of Dar es Salaam, Tanzania, the use of larvicides reduced the risk of malaria infection in 5-year-old children [23]. In the rural highlands of western Kenya, a combination of larvicides and insecticide-treated bed nets (ITNs) decreased new malaria infections by a factor of two compared to ITNs alone [24]. Accordingly, LSM should be considered a crucial part of any integrated mosquito management programme as a complementary means of controlling the spread of mosquito-borne diseases [17,21].

Although the imported dengue epidemic has been effectively controlled, the following five distinct characteristics of Jiaxiang County have led to an increase in mosquito breeding habitats: rich in water resources, concentrated populations, decreased awareness of prevention among residents, unreasonable mosquito-elimination methods and incomplete health management. Mosquito population distributions and disease transmission are also changing. This area has a high incidence of mosquito-borne diseases. Several questions should be addressed. What are the habitat types of local mosquitoes and resistance levels of the dominant mosquito (*Cx*. *p*. *pallens*)? Do current control strategies and measures need to be adjusted? As the above issues have not yet been resolved, it is urgent to investigate local mosquito breeding habitats to understand the relationship between mosquito density and habitat types and the water quality parameters of these breeding habitats, to elucidate the resistance levels of *Cx*. *p*. *pallens* to insecticides and to provide basic data for the scientific prevention and control of mosquitoes.

## Materials and methods

### Description of the study area

Jintun town (35°11’N, 116°06’E) located in the southeast of Jining city, Jiaxiang County, has an area of 92 km^2^ and a population of 67,000. Summer (June to August) is hot and rainy, with a typical warm temperate monsoon climate. The average temperature is 12.8~13.9°C, and the average summer precipitation is 398.5 mm, accounting for 60% of the annual precipitation. Flooding frequently occurs because of concentrated and high-intensity rain events. The Zhuzhaoxin River and its tributaries provide abundant water resources for the region. The main agricultural activities are cultivation of rice, wheat and corn. Every summer and autumn, residents use ditches to divert water into the rice fields [25]. When the dengue fever outbreak occurred in 2017, this area was classified as the core area of the epidemic [3].

### Selection of aquatic habitats

**Fig 1:**
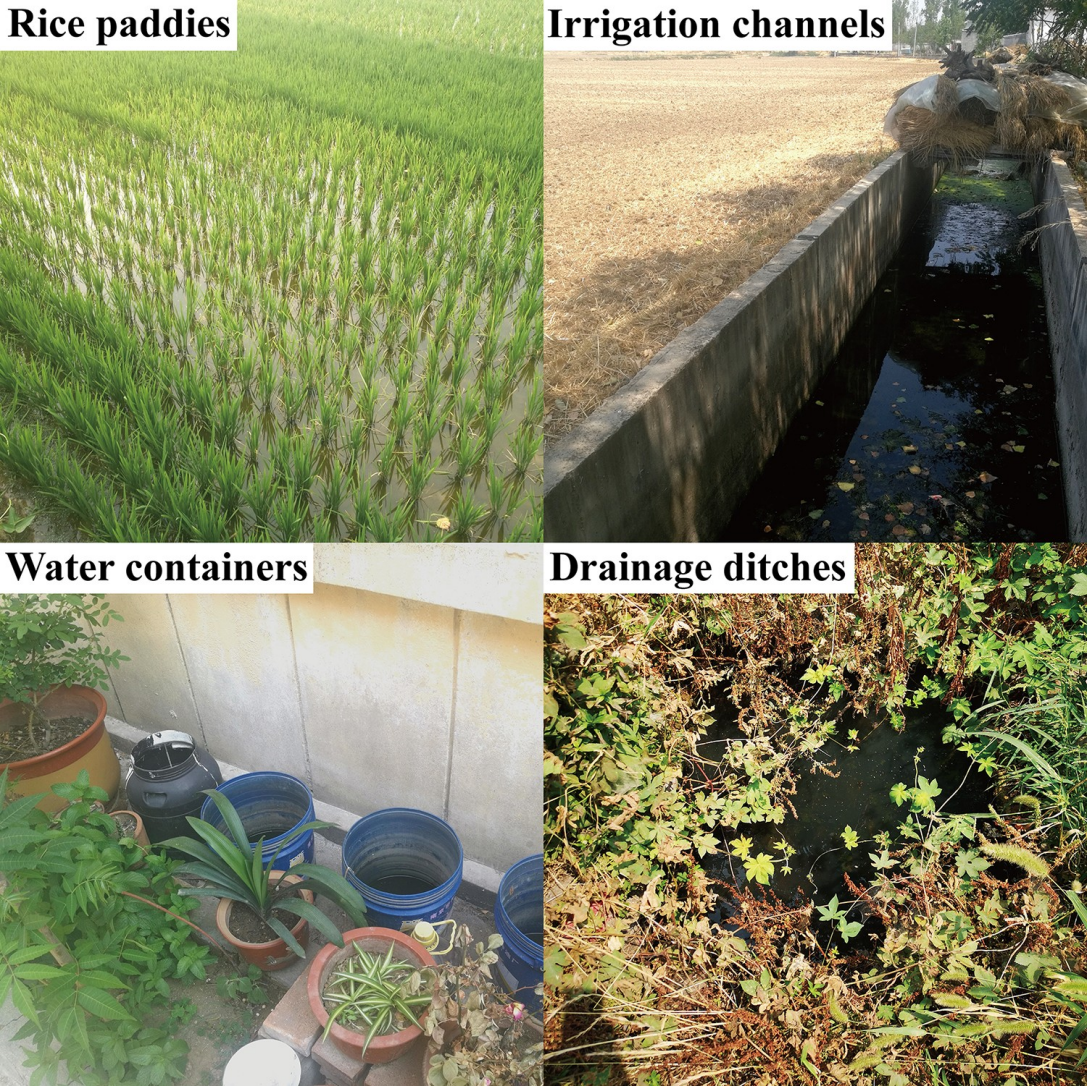
Different types of larval habitats in Jintun town, 2018.

From June to September 2018, larvae were sampled at various sampling locations in Jintun town to identify and evaluate breeding habitats. First, different habitats were observed to determine if larvae were present. The four types of habitats included paddy fields, irrigation channels, water containers and drainage ditches (Fig 1). The habitat was positive for larvae and was included as sampling sites. Each sampling site was given a permanent number for repeated sampling, and 33 sampling sites (5 rice paddies, 5 irrigation channels, 10 water containers and 13 drainage ditches) were identified throughout the survey period. In total, 33 sampling sites were included in each sampling event and sampled 6 times, for 198 sampling events during the survey period. The sampling sites were georeferenced by GPS, and the coordinates (latitude and longitude), habitat type, and vegetation coverage were recorded. Distance from the nearest house was measured by a tape measure. The depth of water in each breeding habitat was obtained by lowering a meter rule to the bottom of the habitat at three sites and then calculating the mean. Water temperature, DO, pH and ammonia nitrogen were recorded using a YSI Professional plus multiparameter metre (YSI Inc., Yellow Springs, OH, USA). All physicochemical parameters were determined on-site only during the first sampling event.

### Larval sampling and identification

A standard dipper (~500 ml) was used to collect water samples from the breeding habitats, collecting three samples from each site. The larval and pupal collection was conducted every two weeks from 9:00 to 13:00 every day. The density of mosquito larvae was calculated by the mean number of larvae per dip sample. The larvae were first classified macroscopically. If the respiratory tube was slender and at a certain angle to the water surface, they were likely *Culex*. If the larvae floated horizontal to the surface of the water, they were likely *Anopheles*. If the respiratory tube was short and at an angle to the water surface, they were likely *Aedes* [26]. The larvae were placed in a specimen box (length = 22 cm, width = 15 cm, height = 15 cm) and transferred to the laboratory, where they were fed pork liver powder and yeast powder until species identification and classification counting after emergence [27]. For the bioassays of larvae is generate by C*x*. *p*. *pallens* adult were fed 10% sucrose solution and blood-with defibrinated sheep blood using a Hemotek unit [5].

### Bioassays

Larval bioassays were performed according to World Health Organization guidelines [28]. DDVP, propoxur, cypermethrin, deltamethrin and *Bti* have been used extensively in China [29]. Adults of *Cx*. *p*. *pallens*, which was the dominant species at each sampling site, were pooled together from all habitat types to produce larvae. We selected F1-generation third-instar larvae of *Cx*. *p*. *pallens* as test specimens. Because the number of collected larvae was too low or no susceptible strain was available, bioassays were not conducted for other mosquito species. Initially, a pre-experiment was conducted regarding the selection of test concentrations of each insecticide. The larvae were exposed to a wide range of test concentrations to determine mortality, and then a narrower concentration range was selected as accurate test concentrations for bioassays [30]. The original insecticides to be tested were formulated into a stock solution and then diluted into five different test concentrations: DDVP (0.125, 0.25, 0.5, 1, 2) mg/l; propoxur (0.28, 0.4, 0.6, 0.9, 1.35) mg/l; cypermethrin (5, 10, 20, 40, 80) μg/l; deltamethrin (2.5, 5, 10, 20, 40) μg/l; *Bti* (0.5, 1, 2, 4, 8, 16) mg/l, with repeated 3 times per concentration. Twenty-five larvae were placed in 100 ml of the different concentrations of insecticides and distilled water. The number of deaths after the larvae were exposed to the insecticide for 24 h was observed and recorded. If a larva did not react when stimulated with a glass rod, it was considered dead. The larvae collected in the field were treated with distilled water, and if the mortality rate was greater than 20%, the experimental data for that group were invalidated. Susceptible laboratory-reared *Cx*. *p*. *pallens* (from the Shandong Center for Disease Control and Prevention; successive generations were cultured in the laboratory, protected from contact with insecticides for 20 years) larvae were placed in plastic cups filled with distilled water or insecticide as a reference. No food was provided. A 12-L:12-D photoperiod, 70%~ 80% relative humidity, and temperature of 28°C were maintained in the laboratory during all bioassays.

### Ethical considerations

No specific licence was required for conducting the field studies. Prior to the start of larval sampling, the town government and residents orally approved all local research sites during assembly of the villagers. We also confirmed that these sites did not involve endangered or protected species.

### Statistical analyses

For statistical calculations, the LC_50_ / LC_95_ was estimated with a log-probit model, and resistance ratios (RR_50_ and RR_95_) were calculated as follows: RR_50_ = field-strain LC_50_ / susceptible-strain LC_50;_ RR_95_ = field-strain LC_95_ / susceptible-strain LC_95_. RRs were classified according to the following categories: susceptibility (RR_50_<3), decreased susceptibility (3<RR_50_<5), low resistance (5<RR_50_<10), moderate resistance (10<RR_50_<40), high resistance (40<RR_50_<160) and very high resistance (RR_50_>160) [31–33]. Generalized linear mixed model (GLMM), with collection site as the random effect variable, was used to test for effects of the collection date and habitat type on the total mosquito abundance and *Cx*. *p*. *pallens* abundance. Univariate analyses of variance (UNIANOVA) for significant interaction terms with post-hoc comparisons were performed by comparing mosquito abundance. Spearman correlation analysis was used to examine the relationship of mosquito larval densities with the physicochemical characteristics adjusted by habitat type. SPSS software (Version 19 for Windows, SPSS Inc., Chicago, IL) was used for the statistical analyses. All statistical analyses were based on a 5% significance level.

## Results

A total of 7,815 mosquitoes, comprising 4 genera and 7 species, were collected from 33 sampling sites in the 4 habitats (Table 1). The types and percentages were as follows: *Cx*. *p*. *pallens* 5,336/68.28%; *Cx*. *tritaeniorhynchus* 1,708/21.85%; *An*. *sinensis* 399/5.11%; *Ar*. *obturbans* 213/2.72%; *Aedes albopictus* 124/1.59%; and other species (*Cx*. *bitaeniorhynchus* and *Cx*. *Halifaxii*) 35/0.45%. *Cx*. *p*. *pallens* was collected in all four habitats. *Cx*. *tritaeniorhynchus* and *An*. *sinensis* were found only in paddy fields and irrigation canals. *Cx*. *bitaeniorhynchus* was found only in irrigation canals, *Ar*. *subalbatus* and *Cx*. *halifaxii* only in drainage ditches, and *Ae*. *albopictus* only in water containers.

**Table 1:** Species distribution and composition^a^.

Mosquito Species	Habitat types					
	Rice paddies (5)	Irrigation channels (5)	Water containers (10)	Drainage ditches (13)	n	%
*Cx*. *p*. *pallens*	219	976	607	3534	5336	68.28%
*Cx*. *tritaeniorhynchus*	1063	645	-	-	1708	21.85%
*An*. *sinensis*	21	378	-	-	399	5.11%
*Ar*. *subalbatus*	-	-	-	213	213	2.72%
*Ae*. *albopictus*	-	-	124		124	1.59%
other^b^		13	-	22	35	0.45%

^a^The data are the number of adult mosquitoes after emergence.

^b^Mosquito species as ‘‘other” included *Cx*. *bitaeniorhynchus* and *Cx*. *halifaxii*.

The results of GLMM analysis showed that there is significant interaction between collection date and habitat type for both total mosquito abundance (*F* = 2.739 *p* = 0.001) and for *Cx*. *p*. *pallens* abundance (*F* = 2.515 *p* = 0.002) (Table 2). Furthermore, for both the total mosquito abundance and the *Cx*. *p*. *pallens* abundance, in rice paddies and irrigation channels, it was highest for mosquitoes sampled on August 21, in water containers, it was highest on August 2, and in drainage ditches, it was highest on July 16. Among them, the total mosquito abundance at each collection date was statistically different in rice paddies and water containers (rice paddies: *F* = 4.030 *p* = 0.009, water containers: *F* = 3.102 *p* = 0.016) (S1 Table).

**Table 2:** Variables from GLMM explaining variation in total mosquito abundance and in *Cx*. *p*. *pallens* abundance.

Response variable	Source	*df*1	*df*2	*F*	*p*
Total mosquito abundance	Corrected model	23	174	4.083	*P*<0.001
	Collected date	5	174	10.795	*p*<0.001
	Habitat type	3	174	4.118	0.007
	Collection date*Habitat type	15	174	2.739	0.001
*Cx*. *p*. *pallens* abundance	Corrected model	23	174	2.877	*p*<0.001
	Collected date	5	174	2.790	0.019
	Habitat type	3	174	4.347	0.006
	Collection date*Habitat type	15	174	2.515	0.002

Among the sampled habitats (Table 3), most of the paddy fields and irrigation canals were far from houses. Water containers such as buckets, water tanks and used tyres were found around residences. Because the water in the paddy fields (28.1±0.4) was directly exposed to sunlight, the water temperature in the paddy fields was the highest. The water depth was deepest in the irrigation channels (0.51±0.04) and shallowest in the water containers (0.17±0.09). The DO of the water in the irrigation canal (151.4±23.0) was highest, followed by the water in the paddy fields (98.8±33.1) and water containers (74.2±11.5); the DO of the water in the drainage ditches (35.7±19.5) was the lowest. The highest pH was observed in the water containers (8.61±0.31) and the lowest pH in the drainage ditches (7.65±0.32). The ammonia nitrogen of the water in the drainage ditches (12.31±7.27) was highest, and the variation in the range was large.

**Table 3:** Means and standard deviations of physicochemical characteristics in different larval habitats in Jintun town, 2018.

*Physicochemical characteristics* (Mean±SD)	Habitat types			
	Rice paddies	Irrigation channels	Water containers	Drainage ditches
Distance from nearest house (m)	68.0±35.6	21.8±21.4	2.8±7.2	10.1±13.2
Water temperature (°C)	28.1±0.4	26.9±2.2	26.8±1.1	25.5±2.4
Water depth (m)	0.19±0.01	0.51±0.04	0.17±0.09	0.20±0.09
DO (%)	98.8±33.1	151.4±23.0	74.2±11.5	35.7±19.5
pH	8.55±0.25	8.10±0.56	8.61±0.31	7.65±0.32
Ammonia nitrogen	0.64±0.07	2.14±0.07	0.49±0.33	12.31±7.27

Spearman correlation analysis was conducted to examine the relationship between mosquito larval densities and physicochemical characteristics (Table 4). The results showed that the density of *Cx*. *tritaeniorhynchus* correlated positively with water depth (*r* = 0.927 *p* = 0.003), the density of *An*. *sinensis* correlated positively with DO (*r* = 0.775 *p* = 0.041) and the density of *Cx*. *p*. *pallens* correlated positively with ammonia nitrogen (*r* = 0.527 *p* = 0.002).

**Table 4:** Spearman correlation coefficient between larval density and physicochemical characteristics of larval habitats adjusted by habitat type in Jintun town, 2018.

		Distance from nearest house	Water temperature	Water depth	DO	pH	Ammonia nitrogen
*Cx*. *p*. *pallens*	*r*	-0.038	0.097	-0.043	-0.269	-0.252	0.527**
	*p*	0.837	0.605	0.818	0.143	0.171	0.002
*Cx*. *tritaeniorhynchus*	*r*	-0.025	-0.071	0.927**	0.714	-0.429	0.393
	*p*	0.589	0.879	0.003	0.071	0.337	0.383
*An*. *sinensis*	*r*	-0.482	0.270	0.718	0.775*	-0.072	0.667
	*p*	0.274	0.558	0.069	0.041	0.878	0.102
*Ar*. *subalbatus*	*r*	-0.564	0.300	-0.600	-0.700	-0.100	0.300
	*p*	0.322	0.624	0.285	0.188	0.873	0.624
*Ae*. *albopictus*	*r*	0.671	0.410	0.600	-0.500	0.200	-0.700
	*p*	0.215	0.493	0.285	0.391	0.747	0.188

*Significant at the 0.05 level

**Significant at the 0.01 level

The RR_50_ values of *Cx*. *p*. *pallens* to DDVP, propoxur, cypermethrin, deltamethrin, and *Bti* were 19.76, 4.60, 438.00, 351.50 and 6.74, respectively. There was a very high level of resistance to cypermethrin and deltamethrin, a moderate level of resistance to DDVP, a low level of resistance to *Bti* and decreased susceptibility to propoxur (Table 5).

**Table 5:** Resistance of *Cx*. *p*. *pallens* to five insecticides in Jintun town, 2018.

Insecticides	Strain	Regression equation	LC_50_ (LCI~UCI)	LC_95_ (LCI~UCI)	RR_50_	RR_95_
DDVP	Jintun strain	Y = 4.5802+1.3478x	2.0486 (1.1720~3.5809)	34.0325 (27.6720~40.1214)	19.76	84.07
	Sensitive strain	Y = 7.7367+2.7800x	0.1037 (0.0884~0.1215)	0.4048 (0.3517~0.4627)	1.00	1.00
Propoxur	Jintun strain	Y = 6.1686+4.0291x	0.5128 (0.4599~0.5719)	1.3129 (1.1506~1.5420)	4.60	4.33
	Sensitive strain	Y = 7.0064+2.1049x	0.1114 (0.0879~0.1412)	0.3034 (0.5753~0.7834)	1.00	1.00
Cypermethrin	Jintun strain	Y = 6.0196+1.1569x	0.1314 (0.0578~0.2988)	3.4712 (2.8734~3.9912)	438.00	2892.67
	Sensitive strain	Y = 14.3177+2.6094x	0.0003 (0.0002~0.0003)	0.0012 (0.0010~0.0015)	1.00	1.00
Deltamethrin	Jintun strain	Y = 6.4147+1.2269x	0.0703 (0.0313–0.1579)	1.7356 (1.4205~2.067)	351.50	1446.33
	Sensitive strain	Y = 11.9562+1.8248x	0.0002 (0.0001~0.0002)	0.0012 (0.0009~0.0015)	1.00	1.00
*Bti*	Jintun strain	Y = 3.4927+1.1906x	18.4521 (7.3000~46.6414)	444.2648 (397.3696~495.3459)	6.74	10.56
	Sensitive strain	Y = 4.3942+1.3860x	2.7359 (2.0453~3.6598)	42.0625 (36.4476~48.9803)	1.00	1.00

LC_50_;Lethal concentration 50% mortality, LC_95_;Lethal concentration 95% mortality

LCI; 95% lower confidence interval, UCI; 95% upper confidence interval

## Discussion

This study aimed to elucidate the species composition and spatial distribution of mosquito larvae in Jining City, Jiaxiang County, determine the physicochemical characteristics of breeding sites and investigate resistance in local dominant populations. These results will help in the future planning and further development of mosquito control programmes that were implemented after the dengue outbreak ended in Jintun town. This survey showed that many types of mosquitoes are present in Jintun town and that the breeding habitats of larvae are complicated. Different mosquito species prefer different breeding habitats. Some physicochemical characteristics of the breeding habitats correlate with mosquito density, and *Cx*. *p*. *pallens* has varying resistance levels to different insecticides.

*Cx*. *tritaeniorhynchus* and *An*. *sinensis* are paddy field mosquitoes that were found in this survey to breed in rice paddies and irrigation canals. Every year in August, deep-water irrigation is required for rice greening and jointing, and the surface water area is supplemented by river water [3,25]. The irrigation channels usually have poor fluidity, and the rice fertilizer often dissolves in the water, causing spirogyra to proliferate; these are favourable breeding conditions for *Cx*. *tritaeniorhynchus* and *An*. *sinensis* [34]. The abundance of *Cx*. *tritaeniorhynchus* is closely related to rice agroecosystems; it is well known that *Cx*. *tritaeniorhynchus* prefers low-lying flooded areas containing grasses, and rice paddies are the main habitat of these larvae [35,36]. In this study, *Cx*. *tritaeniorhynchus* showed a high density in water bodies of large depths, which is consistent with the findings of Bashar et al. [37]. The purpose of the water in rice paddies is to irrigate the plants, but this will also allow some vectors to multiply. According to statistical data from the Food and Agriculture Organization of the United Nations (FAO), China has the world's second largest paddy rice planting area (30,449,860 hectares in 2016) and faces increasing public health threats [38].

In China, *An*. *sinensis* is considered as the principal vector of malaria [39]. *An*. *sinensis* prefers indoor habitats, and Liu et al. found that the maximum flight distance of *An*. *sinensis* is 400 metres; however, most remain within a radius of 100 metres [40]. Additionally, a water depth of 0.5 ~ 1.0 m may be the ideal breeding depth for *An*. *sinensis* [41]. In this study, the density of *An*. *sinensis* correlated positively with DO. Grillet reported a positive association between DO and the abundance of *An*. *oswaldoi*, whereas Amerasinghe et al. reported a negative association between DO and *An*. *culicifacies* [42,43].

During the study period, *Ar*. *subalbatus* was found in only drainage ditches. *Ar*. *subalbatus*, which is one of the mosquito species confirmed in Shandong Province in the 1980s, is mainly found in heavily polluted drains. Due to its large size and strong adaptability to its environment, it is also a highly competitive mosquito species and thus spreads rapidly [44,45]. *Ar*. *subalbatus* prefers alkaline water and breeds in water with a pH greater than 8.2 [46]. Rajavel reported that among various physicochemical characteristics of septic tank habitats, ammonia nitrogen was the only factor correlating significantly with the density of immature *Ar*. *subalbatus* [47].

As a vector of dengue fever, *Ae*. *albopictus* was found in only water containers such as buckets, water tanks and used tyres, in the present study. *Ae*. *albopictus* is a container breeder with the ability to adapt to a low water content, and these mosquitoes prefer to oviposit in shallow water [13,48]. Although Rao et al. believe that the larval density of *Ae*. *albopictus* correlates positively with pH, and the maximum pH range for this species to survive is 6.5–8 [49,50]. As water containers have a limited amount of water, it is possible to control the pH of the breeding habitat by spraying biological insecticides such as neem oil [51], which is a very effective management method for container-breeding mosquitoes.

As has been noted, *Cx*. *p*. *pallens* is the most common mosquito species in Jining and is a potential vector of WNV. The isolation of WNV from mosquitoes and the prevalence of related viral encephalitis have been reported for the Xinjiang Uyghur Autonomous Region of western China [52,53]. Trade, tourism, and migration between China and other countries have increased the likelihood of WNV prevalence in China and even Jining. According to our GLMM analysis, *Cx*. *p*. *pallens* abundance changed over time in the different habitat types. In July and August, rainfall is abundant with increasing temperature, and rice paddies require deep-water irrigation. These favourable climatic and living conditions promote the rapid growth and reproduction of mosquitoes. Data show that *Cx*. *p*. *pallens* prefers polluted water, with the most extensive breeding habitat [7]. In this survey, several types of breeding habitats were found, and the number of *Cx*. *p*. *pallens* was highest in drainage ditches. The newly developed urban communities in the area are densely populated, indicating that household garbage is increasing; some residents dump garbage into the drainage ditch, which gradually becomes blocked, resulting in increased water accumulation and creating an ideal breeding ground for mosquitoes [54]. The density of *Cx*. *p*. *pallens* larvae correlated significantly and positively with ammonia nitrogen. Larvae prefer water bodies with a certain level of pollution, and organic matter in the water body is a necessary condition for the growth of larvae [55]. However, when excessive ammonia nitrogen is discharged into the water body, eutrophication occurs, producing toxins. Overall, the odour of the water body affects mosquito oviposition and larval growth and can inhibit an increase in mosquito density.

Although the chances of mosquitoes being exposed to insecticides differ among habitats, to test the overall resistance level of Jintun town, all third-instar *Cx*. *p*. *pallens* individuals were subjected to bioassays, regardless of habitat type. The results of this survey show that *Cx*. *p*. *pallens* has varying degrees of resistance to various insecticides. This may be related to the long and heavy use of insecticides in various areas to control mosquito density and interrupt transmission routes after the outbreak of dengue fever last year. When arboviral disease is prevalent, chemical insecticides can quickly kill mosquitoes and control the spread of the disease [56]. This result may also be related to the unreasonable use (the range of residual spray is large, and insecticides with a high level of resistance are used) of insecticides. Due to the serious pest damage to agriculture, insecticides are used heavily in this area, with several rounds of spraying conducted in a growing season. Since the 1980s, pyrethroid has been widely used to control agricultural pests [57]. Other insecticides, such as carbamate and organophosphorus, have also been used in the study area since the 1990s [5]. In recent years, pyrethroids have been widely used in Jining under the background of created national health cities. After the Jintun town dengue outbreak, the local government used Chuangweineng^®^ (lambda-cyhalothrin 1%, phoxim 20%), Abate^®^ (temephos 1%), Fendona^®^ (alphacypermethrin 5%) and other insecticides to rapidly reduce mosquito density. Pyrethroid insecticides are highly effective, have low toxicity, and easily decompose in the environment; therefore, they are widely used for the prevention and control of mosquito-borne diseases [58]. Nonetheless, pyrethroid resistance has become the greatest obstacle in the control of mosquito-borne diseases. Moreover, studies have shown that fitness costs associated with insecticide resistance may influence vector-borne disease transmission either by directly reducing the mosquito life span and fecundity, altering mating behaviours or impairing parasite development inside vectors [59–62]. Regardless, the extent to which resistance to insecticides interferes with the impact of mosquito-borne diseases in Jintun town is still unknown. In 2001, the RR_50_ of *Cx*. *p*. *pallens* to cypermethrin and deltamethrin was 27.97 and 12.00, respectively, in the Dongping Lake area of Shandong Province [63]; however, the results of an investigation in 2018 showed that the RR_50_ of these two insecticides reached 83.00 and 106.00 [64], greatly increasing. Liu et al. studied the 20-year change in *Cx*. *p*. *pallens* resistance in Shandong Province and found that resistance to deltamethrin continued to rise from the 1990s to 2018, even though mosquito resistance to other insecticides (DDVP, propoxur, and acetofenate) remained low [5].

According to the current situation in Jintun town, habitat modification, such as humid irrigation, can be adopted in rice paddies; irrigation channels can be covered with cement slabs, effectively controlling the density of *Cx*. *bitaeniorhynchus*, *An*. *sinensis* and other mosquito larvae growing in rice paddies and irrigation channels [17,34]. Additionally, larvivorous fish can be introduced into rice fields to eat mosquito larvae and pupae [65]. Habitat manipulation, such as efforts by the government, should involve dredging of drainage ditches and sewers and cleaning up the garbage that blocks waterways. As water containers are relatively discrete, it is necessary to regularly eliminate aquatic environments, such as turning over such containers to empty the water. All these measures are aimed at preventing *Cx*. *p*. *pallens* or other mosquitoes from ovipositing and producing larvae. Furthermore, we should attempt to use biological insecticides and biological control strategies to reduce the frequency and range of use of pyrethroids, appropriately increase the frequency and range of propoxur, *Bti*, DDVP, and adhere to the rational use of insecticides. Thus, the development of mosquito resistance can be delayed by correct rotation of insecticides with different mechanisms.

It should be noted that there is a limitation of this study. At each sampling site, we measured physicochemical characteristics once while collecting larvae once every half month. Based on the determination of the variation trend of physicochemical characteristics, the possibility of a relationship between larval habitat physicochemical characteristics and larval density fluctuation was established, which has further research importance. Additionally, due to the biological differences between species, as well as the adaptability of the same species to different environments, more research is needed to investigate species composition and habitats in other parts of the Jining, to understand the factors influencing the mosquito ecology, and to obtain reliable basic information about mosquito larvae habitat characteristics. Such information may eventually be used for mosquito control programmes.

## Conclusion

In conclusion, based on the findings of the study, the breeding habitats in Jintun town are extensive, and the physicochemical characteristics of breeding habitats, including water depth, DO, and ammonia nitrogen, may determine the distribution and density of mosquitoes in the area. In areas with high mosquito-borne disease prevalence, intervention measures to effectively control mosquito larvae should focus on water body parameters that are preferred by mosquitoes. Different LSM (habitat modification, habitat manipulation, biological control and larvicide) can be applied in different habitats. *Cx*. *p*. *pallens* is the dominant local species in Jintun town, and its resistance to pyrethroids has reached a very high level. This research aimed to understand mosquito species distributions within aquatic habitats and the efficacy of larval control methods, including the use of insecticides, with increasing resistance.

## Supporting information

S1 Data (XLS)

S2 Data (XLS)

S3 Data (DOCX)

S4 Data (SAV)

S5 Data (SPV)

S1 Table The results of UNIANOVA with post-hoc comparisons. (DOCX)
