# Supplementary material for: Investigation of mosquito larval habitats and insecticide resistance in an area with a high incidence of mosquito-borne diseases in Jining, Shandong Province
Source: PLoS One. 2020 Mar 4;15(3):e0229764. doi: 10.1371/journal.pone.0229764 (PMC7055894; doi:10.1371/journal.pone.0229764)

**The main results of the generalized linear mixed model**

**Fixed effects:** collection date, habitat types, interaction effect (collection date * habitat types)

**Random effect:** collection site

**Target：**Total mosquito abundance


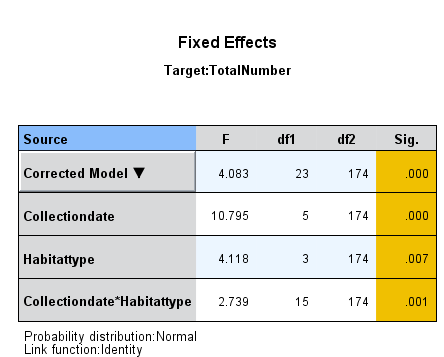


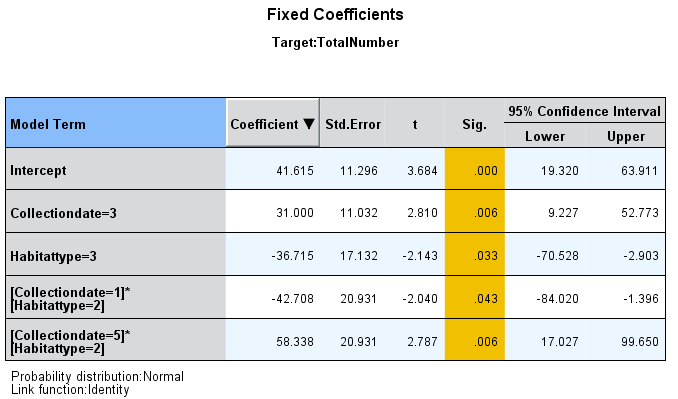


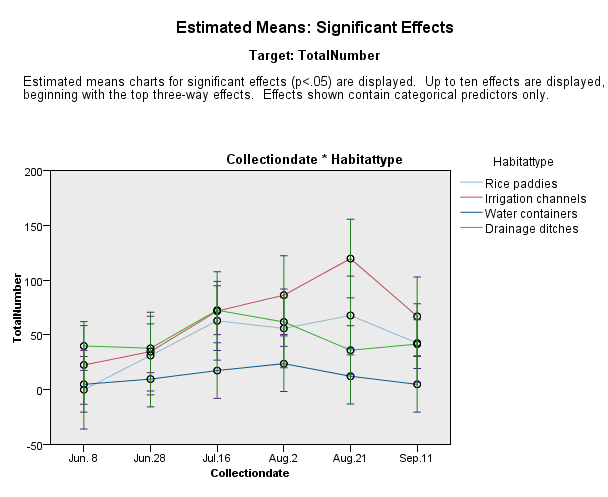


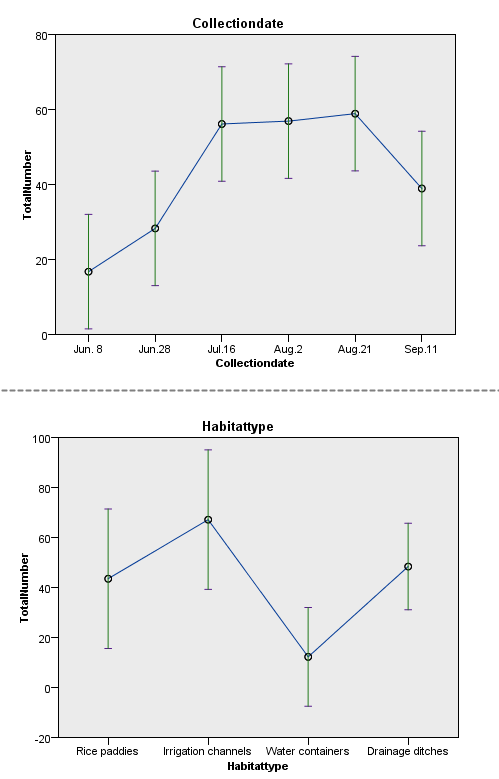


**Target：***Cx. p. pallens* abundance


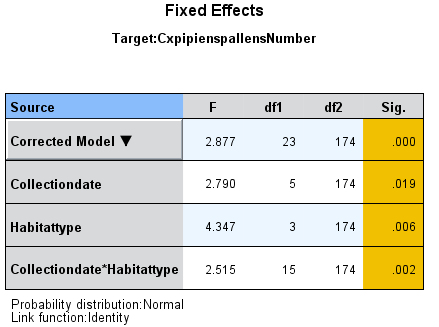


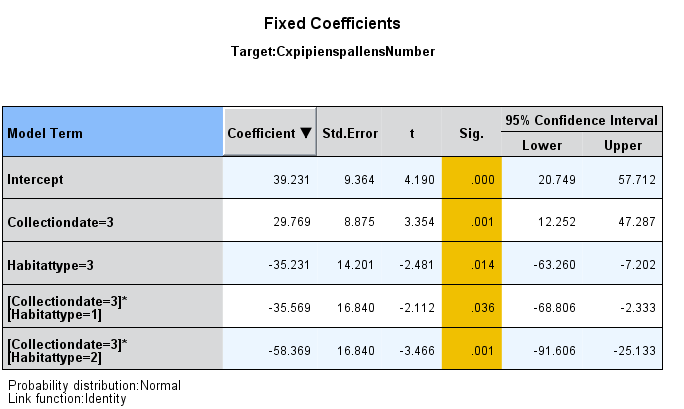


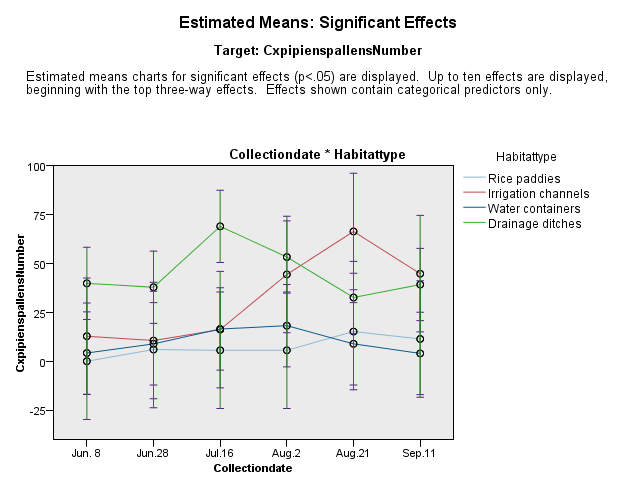


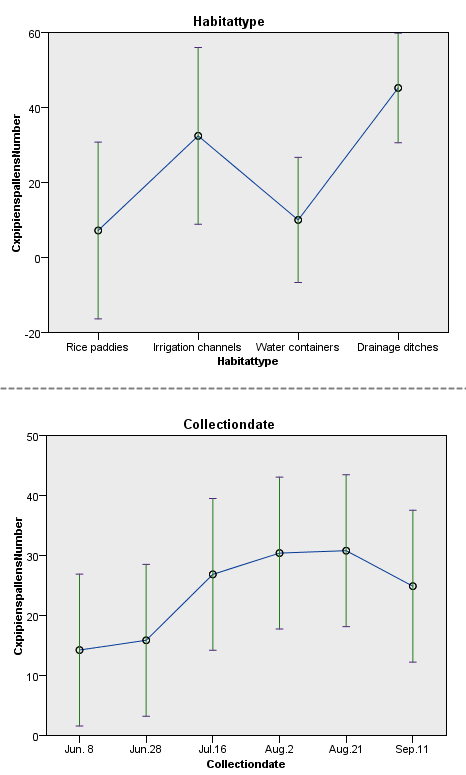

Supplement: S3 Data — (DOCX) [file pone.0229764.s003.docx]
