# Supplementary material for: Investigation of mosquito larval habitats and insecticide resistance in an area with a high incidence of mosquito-borne diseases in Jining, Shandong Province
Source: PLoS One. 2020 Mar 4;15(3):e0229764. doi: 10.1371/journal.pone.0229764 (PMC7055894; doi:10.1371/journal.pone.0229764)
Supplement: S1 Table — (DOCX) [file pone.0229764.s006.docx]

**The results of UNIANOVA with post-hoc comparisons：**

| **Dependent Variable: Total mosquito abundance**   \| **ANOVA** \| \| \| \| \| \| \| \| --- \| --- \| --- \| --- \| --- \| --- \| --- \| \| Habitat type \| \| Sum of Squares \| *df* \| Mean Square \| *F* \| Sig. \| \| Rice paddies \| Between Groups \| 15856.567 \| 5 \| 3171.313 \| 4.030 \| .009 \| \| Within Groups \| 18884.800 \| 24 \| 786.867 \|  \|  \| \| Total \| 34741.367 \| 29 \|  \|  \|  \| \| Irrigation channels \| Between Groups \| 30977.067 \| 5 \| 6195.413 \| 1.973 \| .119 \| \| Within Groups \| 75346.800 \| 24 \| 3139.450 \|  \|  \| \| Total \| 106323.867 \| 29 \|  \|  \|  \| \| Water containers \| Between Groups \| 2754.883 \| 5 \| 550.977 \| 3.102 \| .016 \| \| Within Groups \| 9592.100 \| 54 \| 177.631 \|  \|  \| \| Total \| 12346.983 \| 59 \|  \|  \|  \| \| Drainage ditches \| Between Groups \| 14927.603 \| 5 \| 2985.521 \| 1.163 \| .336 \| \| Within Groups \| 184817.385 \| 72 \| 2566.908 \|  \|  \| \| Total \| 199744.987 \| 77 \|  \|  \|  \|   **Multiple Comparisons** | | | | | | | |
| --- | --- | --- | --- | --- | --- | --- | --- | --- | --- | --- | --- | --- | --- | --- | --- | --- | --- | --- | --- | --- | --- | --- | --- | --- | --- | --- | --- | --- | --- | --- | --- | --- | --- | --- | --- | --- | --- | --- | --- | --- | --- | --- | --- | --- | --- | --- | --- | --- | --- | --- | --- | --- | --- | --- | --- | --- | --- | --- | --- | --- | --- | --- | --- | --- | --- | --- | --- | --- | --- | --- | --- | --- | --- | --- | --- | --- | --- | --- | --- | --- | --- | --- | --- | --- | --- | --- | --- | --- | --- | --- | --- | --- | --- | --- | --- | --- | --- |
| LSD | | | | | | | |
| Habitat type | Collection date (I) | Collection date (J) | Mean Difference (I-J) | Std. Error | Sig. | 95% Confidence Interval | |
|  |  |  |  |  |  | Lower Bound | Upper Bound |
| Rice paddies | Aug.21 | Jun. 8 | 67.800^*^ | 17.741 | .001 | 31.18 | 104.42 |
|  |  | Jun.28 | 36.600 | 17.741 | .050 | -.02 | 73.22 |
|  |  | Jul.16 | 4.800 | 17.741 | .789 | -31.82 | 41.42 |
|  |  | Aug.2 | 11.800 | 17.741 | .512 | -24.82 | 48.42 |
|  |  | Sep.11 | 25.200 | 17.741 | .168 | -11.42 | 61.82 |
| Irrigation channels | Aug.21 | Jun. 8 | 97.200^*^ | 35.437 | .011 | 24.06 | 170.34 |
|  |  | Jun.28 | 85.000^*^ | 35.437 | .025 | 11.86 | 158.14 |
|  |  | Jul.16 | 48.000 | 35.437 | .188 | -25.14 | 121.14 |
|  |  | Aug.2 | 33.400 | 35.437 | .355 | -39.74 | 106.54 |
|  |  | Sep.11 | 52.800 | 35.437 | .149 | -20.34 | 125.94 |
| Water containers | Aug.2 | Jun. 8 | 18.900^*^ | 5.960 | .003 | 6.95 | 30.85 |
|  |  | Jun.28 | 14.100^*^ | 5.960 | .022 | 2.15 | 26.05 |
|  |  | Jul.16 | 6.300 | 5.960 | .295 | -5.65 | 18.25 |
|  |  | Aug.21 | 11.500 | 5.960 | .059 | -.45 | 23.45 |
|  |  | Sep.11 | 18.900^*^ | 5.960 | .003 | 6.95 | 30.85 |
| Drainage ditches | Jul.16 | Jun. 8 | 32.692 | 19.872 | .104 | -6.92 | 72.31 |
|  |  | Jun.28 | 34.769 | 19.872 | .084 | -4.85 | 74.38 |
|  |  | Aug.2 | 10.769 | 19.872 | .590 | -28.85 | 50.38 |
|  |  | Aug.21 | 36.538 | 19.872 | .070 | -3.08 | 76.15 |
|  |  | Sep.11 | 31.000 | 19.872 | .123 | -8.61 | 70.61 |
| *. The mean difference is significant at the 0.05 level. | | | | | | | |

**Dependent Variable: *Cx. p. pallens* abundance**

| **ANOVA** | | | | | | |
| --- | --- | --- | --- | --- | --- | --- |
| Habitat | | Sum of Squares | *df* | Mean Square | *F* | Sig. |
| Rice paddies | Between Groups | 699.900 | 5 | 139.980 | 1.502 | .226 |
|  | Within Groups | 2236.400 | 24 | 93.183 |  |  |
|  | Total | 2936.300 | 29 |  |  |  |
| Irrigation channels | Between Groups | 12877.467 | 5 | 2575.493 | 2.320 | .075 |
|  | Within Groups | 26640.000 | 24 | 1110.000 |  |  |
|  | Total | 39517.467 | 29 |  |  |  |
| Water containers | Between Groups | 1814.683 | 5 | 362.937 | 2.352 | .053 |
|  | Within Groups | 8333.500 | 54 | 154.324 |  |  |
|  | Total | 10148.183 | 59 |  |  |  |
| Drainage ditches | Between Groups | 11815.077 | 5 | 2363.015 | 1.056 | .392 |
|  | Within Groups | 161131.538 | 72 | 2237.938 |  |  |
|  | Total | 172946.615 | 77 |  |  |  |

| **Multiple Comparisons** | | | | | | | |
| --- | --- | --- | --- | --- | --- | --- | --- |
| LSD | | | | | | | |
| Habitat type | Collection date (I) | Collection date (J) | Mean Difference (I-J) | Std. Error | Sig. | 95% Confidence Interval | |
|  |  |  |  |  |  | Lower Bound | Upper Bound |
| Rice paddies | Aug.21 | Jun. 8 | 15.200^*^ | 6.105 | .020 | 2.60 | 27.80 |
|  |  | Jun.28 | 9.200 | 6.105 | .145 | -3.40 | 21.80 |
|  |  | Jul.16 | 9.600 | 6.105 | .129 | -3.00 | 22.20 |
|  |  | Aug.2 | 9.600 | 6.105 | .129 | -3.00 | 22.20 |
|  |  | Sep.11 | 3.800 | 6.105 | .540 | -8.80 | 16.40 |
| Irrigation channels | Aug.21 | Jun. 8 | 53.600^*^ | 21.071 | .018 | 10.11 | 97.09 |
|  |  | Jun.28 | 55.800^*^ | 21.071 | .014 | 12.31 | 99.29 |
|  |  | Jul.16 | 50.200^*^ | 21.071 | .025 | 6.71 | 93.69 |
|  |  | Aug.2 | 22.000 | 21.071 | .307 | -21.49 | 65.49 |
|  |  | Sep.11 | 21.600 | 21.071 | .316 | -21.89 | 65.09 |
| Water containers | Aug.2 | Jun. 8 | 14.000^*^ | 5.556 | .015 | 2.86 | 25.14 |
|  |  | Jun.28 | 9.300 | 5.556 | .100 | -1.84 | 20.44 |
|  |  | Jul.16 | 1.700 | 5.556 | .761 | -9.44 | 12.84 |
|  |  | Aug.21 | 9.300 | 5.556 | .100 | -1.84 | 20.44 |
|  |  | Sep.11 | 14.200^*^ | 5.556 | .013 | 3.06 | 25.34 |
| Drainage ditches | Jul.16 | Jun. 8 | 29.154 | 18.555 | .121 | -7.84 | 66.14 |
|  |  | Jun.28 | 31.154 | 18.555 | .097 | -5.84 | 68.14 |
|  |  | Aug.2 | 15.692 | 18.555 | .401 | -21.30 | 52.68 |
|  |  | Aug.21 | 36.385 | 18.555 | .054 | -.60 | 73.37 |
|  |  | Sep.11 | 29.769 | 18.555 | .113 | -7.22 | 66.76 |
| *. The mean difference is significant at the 0.05 level. | | | | | | | |
